# Characteristics of gut microbiota in high-methylated colorectal cancer

**DOI:** 10.1007/s10147-025-02812-3

**Published:** 2025-06-16

**Authors:** Tatsushi Saito, Hideaki Karasawa, Kota Ouchi, Tomoyuki Ono, Taiki Kajiwara, Atsushi Kohyama, Wakako Ikeda-Ohtsubo, Shinji Fukuda, Fumiyoshi Fujishima, Yohei Ozawa, Hideyuki Suzuki, Kazuhiro Watanabe, Chikashi Ishioka, Takashi Kamei, Shinobu Ohnuma, Takaaki Abe, Michiaki Unno

**Affiliations:** 1 Department of Surgery, Tohoku University Graduate School of Medicine, 1‐1 Seiryo-machi, Aoba-Ku, Sendai, 980-8574 Japan; 2 Department of Oncology, Tohoku University Graduate School of Medicine, 1‐1 Seiryo-machi, Aoba-Ku, Sendai, 980-8574 Japan; 3 Food and Feed Immunology Group, Laboratory of Animal Food Function, Graduate School of Agricultural Science, Tohoku University, Sendai, 980-8572 Japan; 4 Institute for Advanced Biosciences, Keio University, 246-2 Kakuganji, Mizukami, Tsuruoka, 997-0052 Japan; 5 Innovative Microbiome Therapy Research Center, Juntendo University Graduate School of Medicine, 2-1-1 Hongo, Bunkyo-Ku, Tokyo, 113-8421 Japan; 6 Gut Environmental Design Group, Kanagawa Institute of Industrial Science and Technology, 3-25-13 Tono-Machi, Kawasaki-Ku, Kawasaki, 210-0821 Japan; 7 Transborder Medical Research Center, University of Tsukuba, 1-1-1 Tennoudai, Tsukuba, 305-8577 Japan; 8 Department of Pathology, Tohoku University Hospital, 1‐1 Seiryo-machi, Aoba-Ku, Sendai, 980-8574 Japan; 9 Division of Diagnostic Pathology, Tohoku Medical and Pharmaceutical University, Sendai, 983-8512 Japan; 10 Department of Biomedical Engineering Regenerative and Biomedical Engineering Medical Science, Tohoku University Graduate School of Biomedical Engineering, 6-6 Aoba, Aoba-Ku, Sendai, 980-8579 Japan

**Keywords:** Colorectal cancer, High-methylated colorectal cancer, CpG island methylator phenotype, Fusobacterium nucleatum

## Abstract

**Background:**

Epigenetic alterations, including DNA methylation, significantly contribute to colorectal cancer (CRC); the gut microbiota is also involved. However, studies on the possible role of microbiota in DNA methylation are limited. This study investigates the association between gut microbiota composition and high-methylated CRC (HMCC).

**Methods:**

Fecal and tumor tissue samples were collected from 86 patients with sporadic CRC. HMCC was defined based on the methylation status of 16 CpG sites of tumor-derived genomic DNA. 16S rRNA gene sequencing was performed to reveal the composition of the gut microbiota. The load of *Fusobacterium nucleatum (F. nucleatum)* in tumor tissues was assessed using quantitative polymerase chain reaction (qPCR).

**Results:**

HMCC was identified in 21 patients, whereas 65 were classified as having low-methylated CRC (LMCC). HMCC was significantly associated with proximal location, large diameter, undifferentiated histology, and high frequency of *BRAF* mutation. In gut microbial analyses, the relative abundances of 84 bacteria, including *F. nucleatum,* were significantly different between HMCC and LMCC. The load of *F. nucleatum* in CRC specimens was significantly correlated with its relative abundance in fecal samples and tended to be enriched in HMCC tissues.

**Conclusions:**

This study characterizes the gut microbiota profile in HMCC and suggests that bacteria, such as *F. nucleatum,* may contribute to HMCC pathogenesis through DNA methylation. Further studies are needed to determine whether the microbiome acts as a promoter or bystander in HMCC development.

## Introduction

Colorectal cancer (CRC) is the third most prevalent cancer worldwide [1], with its incidence more than doubling between 1990 and 2019 [2]. This rise is attributed to factors, such as Westernized diets and aging populations, and the trend is expected to continue [3]. Although the CRC incidence rate has decreased in high-income North American countries and Australasia due to widespread colonoscopy screening, the burden is shifting toward low- and middle-income countries [2]. This underscores the urgent need for improved CRC preventive strategies.

Advancements in gut microbiota research have revealed its role in various diseases, immunomodulation, and health maintenance [4, 5]. Colorectal diseases, including CRC, reduce the diversity of fecal microbiota [6, 7]. Specific bacteria, such as *Fusobacterium nucleatum (F. nucleatum), Escherichia coli (E. coli)*, and *Bacteroides fragilis* (*B. fragilis)*, contribute to CRC pathogenesis [4]. Although the primary focus has been on bacterial contributions through chronic inflammatory and immune response modulations [8, 9], epigenetic effects, such as DNA methylation, also play a role [10]. Furthermore, bacterial metabolites and biofilms are potential contributors to CRC development [11–13]. Thus, gut microbiota research shows promise in advancing CRC prevention and diagnostics.

CRC develops through multiple carcinogenic pathways. The adenoma-carcinoma sequence (ACS), characterized by the accumulation of genomic mutations, is a well-established pathway. Recently, the serrated pathway, which arises from serrated precursor lesions, has been recognized [14]. The serrated pathway is characterized by epigenomic alterations, in contrast to ACS. Genome-wide hypermethylation in the DNA CpG sites, CpG island methylator phenotype (CIMP), is often observed in this pathway [15, 16]. CIMP causes mismatch repair deficiency (dMMR) by gene silencing via DNA methylation, and accumulation of dMMR-induced replication errors leads to microsatellite instability (MSI) [15, 17, 18]. CIMP- and MSI-positive CRCs share unique clinicopathological features, such as proximal location, poor differentiation, and poor prognosis [15, 16, 19–21].

CIMP is diagnosed by evaluating the methylation status of specific gene CpG regions, serving as CIMP markers depending on the number of methylated genes, although consensus on which genes remains lacking [15, 22]. Therefore, we investigated the genome-wide methylation status of tumor tissue DNA in patients with metastatic CRC and found that high-methylated CRC (HMCC) cases had a significantly lower response rate to anti-epidermal growth factor receptor (EGFR) therapy compared to low-methylated CRC (LMCC) cases [23]. In 97 metastatic CRC cases, the HMCC subgroup predominantly included CIMP-positive cases identified using various CIMP markers, alongside multiple CIMP-negative cases [23]. These HMCC cases exhibited characteristics similar to CIMP-positive cases, such as proximal location, poor differentiation, and poor prognosis [23, 24]. Thus, we considered HMCC as a more accurate and comprehensive DNA methylation characteristic than CIMP. Then, we identified the optimal set of 16 CpG regions to classify CRC into HMCC and LMCC; this classification was an independent predictive factor and a more accurate biomarker than the primary site of anti-EGFR treatment [24].

Fecal transplantation from patients with CRC to CRC mouse models promoted DNA methylation, suggesting that certain bacteria may induce DNA methylation [25]. Although the potential involvement of the microbiota in DNA methylation has primarily been investigated in the context of CIMP-positive tumors and *F. nucleatum* [10], the association between the microbiota and CIMP remains inconsistent in existing literature [10]. Therefore, we hypothesized that specific gut bacteria induce HMCC carcinogenesis via DNA methylation and conducted this study to elucidate the characteristic gut microbiota in HMCC.

## Patients and methods

### Patients and sample collection

This study included 86 patients with CRC who underwent surgery at Tohoku University Hospital between April 2021 and December 2022, along with 63 healthy adults. To eliminate the influence of genetic factors and chronic inflammatory environments, patients with Lynch syndrome, familial adenomatous polyposis, or inflammatory bowel diseases were excluded. Healthy controls were included only if they had no history of cancer or surgery. The sample sizes of 86 patients and 63 healthy controls were not determined through formal statistical calculations. As this was an exploratory study, we included all eligible participants who met the inclusion criteria during the study period. Written informed consent was obtained from each patient with CRC. This study was approved by the Research Ethics Committee of the Tohoku University Graduate School of Medicine (No. 2020-1-1054). In CRC cases, fecal samples were collected preoperatively, and 5 mm^2^ pieces of tumor tissue were collected from the resected specimens during surgery. Each sample was stored in a − 80 °C deep freezer until DNA extraction. For healthy controls, only fecal samples were collected and preserved under identical conditions.

### Data collection

We collected data on each patient’s age, sex, and primary tumor characteristics (location and pathology). Tumors were categorized according to their primary location as right-sided (cecum to transverse colon) or left-sided (splenic flexure to anorectal junction). Histologically, tumors were classified into two groups: (1) differentiated (papillary and tubular adenocarcinoma) and (2) undifferentiated (poorly differentiated adenocarcinoma, mucinous adenocarcinoma, and signet-ring cell carcinoma). In cases of multiple mixed histological types, the predominant histological type was used.

### DNA methylation analysis

Genomic DNA was extracted from tumor tissues using the QIAamp DNA Mini Kit (QIAGEN, Germany). The concentration and quality of the DNA were evaluated using the NanoDrop One (Thermo Fisher Scientific Inc., USA). Methylation status was analyzed using a modified MethyLight assay, as previously reported [24]. Briefly, the DNA was treated with bisulfite, and real-time quantitative polymerase chain reaction (qPCR) was performed using primer and probe sets targeting the 16 CpG sites. Cases with ≥ 8 methylation-positive sites (out of the 16 sites) were classified as HMCC, whereas those with ≤ 7 methylation-positive sites were classified as LMCC. The cutoff value of ≥ 8 positive methylation markers was determined based on receiver operating characteristic curve analysis in a previous study [24], which identified this threshold as optimal for distinguishing HMCC from LMCC. This criterion was adopted in the present study to ensure consistency with established methods.

### Immunohistochemistry for mismatch repair (MMR) proteins

MMR protein expression was assessed using immunohistochemistry with an Autostainer Link48 (DAKO, Agilent Technologies, Denmark) with the following antibodies: anti-MLH1 (ES05; DAKO), anti-MSH2 (FE11; DAKO), anti-MSH6 (EP49; DAKO), and anti-PMS2 (EP51; DAKO). Tumors were classified as proficient MMR (pMMR) if all four proteins were expressed, and as deficient MMR (dMMR) if at least one protein was absent.

### PCR–reverse sequence-specific oligonucleotide probe (PCR-rSSO)

*The RAS* and *BRAF* gene status in each case was analyzed using the PCR-reverse sequence-specific oligonucleotide probe (PCR-rSSO) method. The MEBGEN RASKET-B KIT (MBL, Japan) was used to identify mutations in exons 2 (codons 12 and 13), 3 (codons 59 and 61), and 4 (codons 117 and 146) of *KRAS* and *NRAS* (*RAS* status) and *BRAF V600E*.

### 16S rRNA gene sequencing

To determine the gut microbiota composition, 16S rRNA gene sequencing was performed. Fecal samples were freeze-dried, crushed, and vigorously shaken with 0.1 mm zirconia/silica beads using a Shake Master (Biomedical Science, Japan). The emulsion was centrifuged at 17,800 × g for 10 min at room temperature, and the bacterial genomic DNA was purified from the aqueous phase using a standard phenol/chloroform/isoamyl alcohol protocol. The V1–V2 region of the 16S rRNA genes was amplified using the bacterial universal primer set Rd1 SP sequence-27Fmod MIX (5′-ACACTCTTTCCCTACACGACGCTCTTCCGATCTNNNNNAGRGTTTGATYMTGGCTCAG-3′), Rd2 SP sequence-338R MIX (5′-GTGACTGGAGTTCAGACGTGTGCTCTTCCGATCTNNNNNTGCTGCCTCCCGTAGGAGT-3′). PCR was performed using Tks Gflex DNA polymerase (Takara, Japan) under the following conditions: initial denaturation at 94 °C for 2 min, followed by 30 cycles at 94 °C for 30 s, 55 °C for 30 s, and 72 °C for 30 s, with a final extension at 72 °C for 5 min. PCR products were purified using Agencourt AMPure XP (Beckman Coulter, Atlanta, GA, USA). The purified products were then further amplified using a primer pair as follows: a forward primer (5′-AATGATACGGCGACCA CCGAGATCTACAC-NNNNNNNN-TATGGTAATTGTAGRGTTTGATYMTGGCTCAG-3′) containing the p5 sequence, unique 8-bp barcode sequence for each sample (indicated from the string of Ns), overhang adapter, and reverse primer (5′-CAAGCAGAAGACGGCATACGAGAT-NNNNNNNN-AGTCAGTCAGCCTGCTGCCTCCCGTAGGAGT-3′) containing the P7 sequence, unique 8-bp barcode sequence for each sample (indicated by the string of Ns), and overhang adapter. After purification using Agencourt AMPure XP kits, the purified products were mixed at approximately equal molar concentrations to generate a 4 nM library pool, after which the final library pool was diluted to 6 pM, including a 10% Phix Control v3 (Illumina, San Diego, California, U.S.A.) spike-in for sequencing. MiSeq sequencing was performed according to the manufacturer’s instructions. In this study, paired-end (2 × 300-bp) sequencing was performed. Sequences were analyzed using the dada2 plug-in in Qiime2 (ver. 2022.2) to remove chimeric and noise sequences. DNA sequences with more than 97% homology in operational taxonomic units (OTUs) were clustered, with one OTU corresponding to one bacterial species. Representative sequences and ASV tables were compared with representative sequences obtained using the feature-classifier plug-in and EzBioCloud 16S database (ChunLab Inc., Korea) for phylogenetic inference.

### Quantitative polymerase chain reaction (qPCR) for F. nucleatum

Genomic DNA was extracted from tumor tissues using the QIAamp DNA Mini Kit (Qiagen, Hilden, Germany). The TaqMan primer/probe set for *nusG*, a specific gene sequence of *F. nucleatum*, and the reference gene *PGT* were used as previously reported [26]. qPCR for *F. nucleatum* with the 2-ΔCt method was performed using the Premix Ex Taq (Perfect Real Time, Takara, Japan). Each reaction contained 100 ng of genomic DNA, 10 uL of 2 × Premix Ex taq, 0.4 uL of 50 × ROX reference Dye, 0.4 uL of forward primer, 0.4 uL of reverse primer, and 0.8 uL of FAM probe, with a 20 uL final reaction volume. The amplification proceeded with one denaturation step at 95 °C for 20 s, 40 cycles of 95 °C for 1 s, and then 60 °C for 20 s. Samples without amplification were assigned a threshold cycle (Ct) of 40 and considered negative. All analyses were performed in duplicate using Step One PlusTM (Applied Biosystems Inc., USA).

### Statistical analyses

Clinicopathological factors were analyzed using the chi-square test, Wilcoxon test, or Fisher’s exact test in JMP Pro 17 (SAS Institute Japan Co., Ltd.). Gut microbial analyses were performed using the R software (version 4.2.3, R Foundation for Statistical Computing, Australia). We used the Mann–Whitney U test to compare the bacterial composition. Alpha diversity (Shannon index) and beta diversity (Bray–Curtis distances) were calculated from rarefied OTU data. Linear regression and permutational multivariate analysis of variance (PERMANOVA) were used for between-group comparisons.

## Results

### Clinicopathological characteristics

The median age of the 86 patients with CRC (56 men and 30 women) was 66 years (25–85 years). DNA methylation was analyzed using a modified MethyLight assay. Thus, 21 patients (24.4%) were classified as having HMCC and 65 (75.6%) as having LMCC. We compared the clinicopathological factors between the two groups (Table 1). HMCC was associated with proximal location (p = 0.04), large diameter (p < 0.01), undifferentiated histology (p < 0.01), lymphatic invasion (p = 0.04), and a high frequency of dMMR and *BRAF* mutations (p = 0.03, p < 0.01, respectively). The 63 healthy controls, including 25 men and 38 women, had a median age of 50 years (24–63 years).

**Table 1:** Association between methylation status and clinicopathological factors in 86 patients with CRC

Characteristics	No. (%)			p value
	All cases (n = 86)	HMCC (n = 21)	LMCC (n = 65)	
Age, median (range), year	68 (25–85)	71 (49–85)	68 (25–84)	0.41
Sex				
Male	56 (65.1)	13 (61.9)	43 (66.2)	0.72
Female	30 (34.9)	8 (38.1)	22 (33.9)	
Tumor location				
Right	22 (25.6)	9 (42.9)	13 (20.0)	0.04
Left	64 (74.4)	12 (57.1)	52 (80.0)	
Size, median (range), mm	35.5 (10–135)	56 (20–97)	30 (10–135)	< 0.01
Histologic differentiation				
Differentiated	81 (94.2)	17 (80.9)	64 (98.5)	< 0.01
Undifferentiated	5 (5.8)	4 (19.1)	1 (1.5)	
Depth of invasion^a^				
Tis + T1 + T2	33 (38.4)	7 (33.3)	26 (40.0)	0.58
T3 + T4	53 (61.6)	14 (66.7)	39 (60.0)	
LN metastasis				
No	49 (57.0)	9 (42.9)	40 (61.5)	0.13
Yes	37 (43.0)	12 (57.1)	25 (38.5)	
Stage^a^				
0 + I + II	46 (53.5)	8 (38.1)	38 (58.5)	0.12
III + IV	40 (46.5)	13 (61.9)	27 (41.5)	
Lymphatic invasion				
No	45 (52.3)	7 (33.3)	38 (58.5)	0.04
Yes	41 (47.7)	14 (66.7)	27 (41.5)	
Venous invasion				
No	18 (20.9)	2 (9.5)	16 (24.6)	0.12
Yes	68 (79.1)	19 (90.5)	49 (75.4)	
MMR status				
pMMR	77 (89.5)	16 (76.2)	61 (93.8)	0.03
dMMR	9 (10.5)	5 (23.8)	4 (6.2)	
*RAS* status				
Wild type	45 (52.3)	10 (47.6)	35 (53.8)	0.62
Mutant	41 (47.7)	11 (52.4)	30 (46.2)	
*BRAF* status				
Wild type	81 (94.2)	16 (76.2)	65 (100)	< 0.01
Mutant	5 (5.8)	5 (23.8)	0 (0.0)	
Use of probiotics				
Yes	4 (4.7)	2 (9.5)	2 (3.1)	0.26
No	82 (95.3)	19 (90.5)	63 (96.1)	
Use of antibiotics				
Yes	4 (4.7)	2 (9.5)	2 (3.1)	0.26
No	82 (95.3)	19 (90.5)	63 (96.1)	

*HMCC* high-methylated colorectal cancer, *LMCC* low-methylated colorectal cancer, *MMR* mismatch repair gene function, *pMMR* proficient MMR, *dMMR* deficient MMR

^a^Based on TNM classification

### Gut microbial analyses

**Fig. 1:**
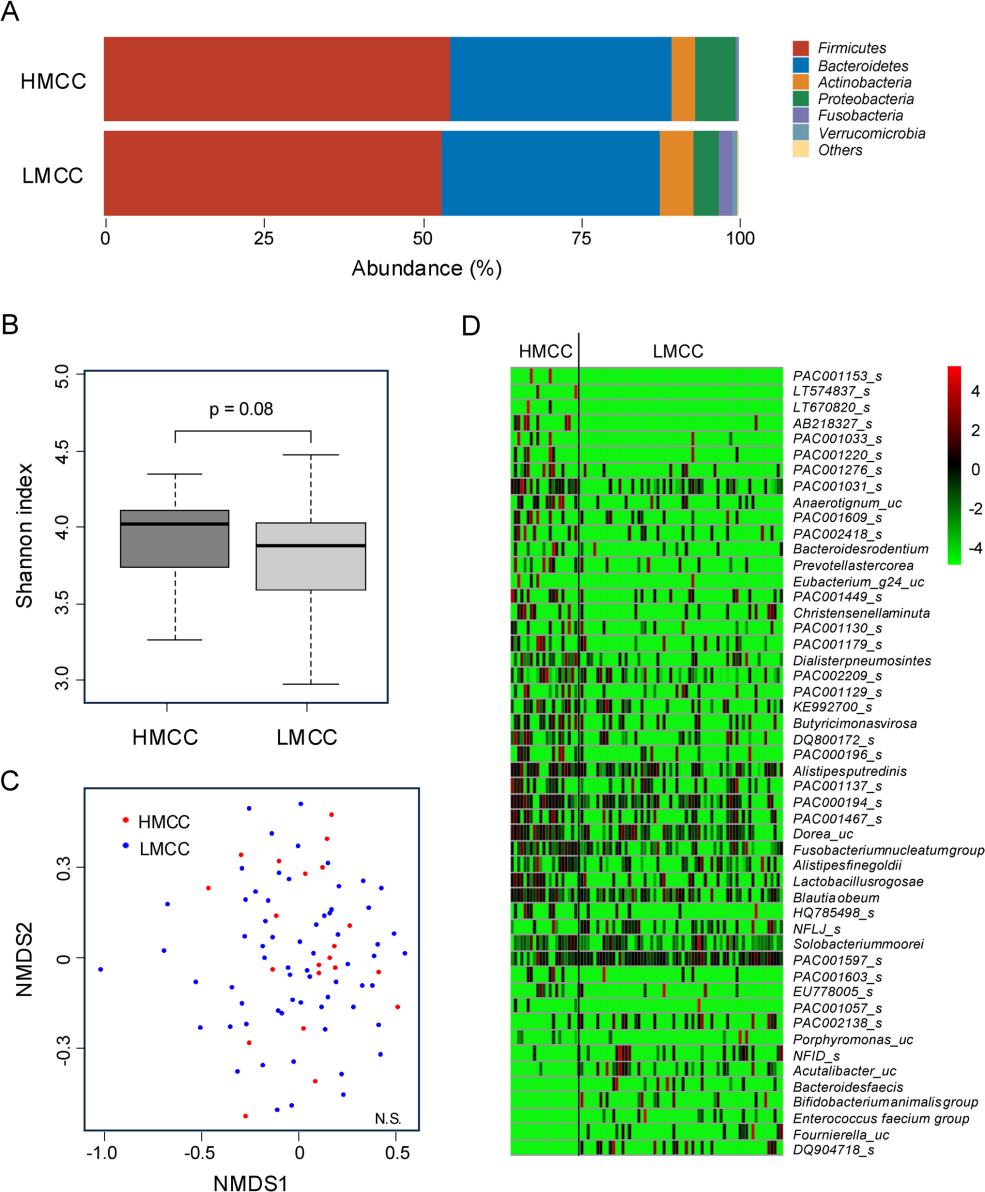
Comparison of fecal bacterial composition between HMCC and LMCC groups. **A** Bar chart depicting the mean relative abundances of the top six phyla in the HMCC and LMCC groups; **B** comparison of Shannon indices indicating no significant differences between the two groups (linear regression, p = 0.07); **C** Bray–Curtis distances of beta diversity of the two groups visualized using nonmetric multidimensional scaling (NMDS). The two axes (NMDS1 and NMDS2) represent the two most significant dimensions of variation among the samples. PERMANOVA revealed no significant differences between the two groups (p = 0.32); **D** heatmap showing the log-transformed relative abundance normalized by the mean abundance of the top 50 most abundant bacteria in all cases, with significant differences between the two groups

**Fig. 2:**
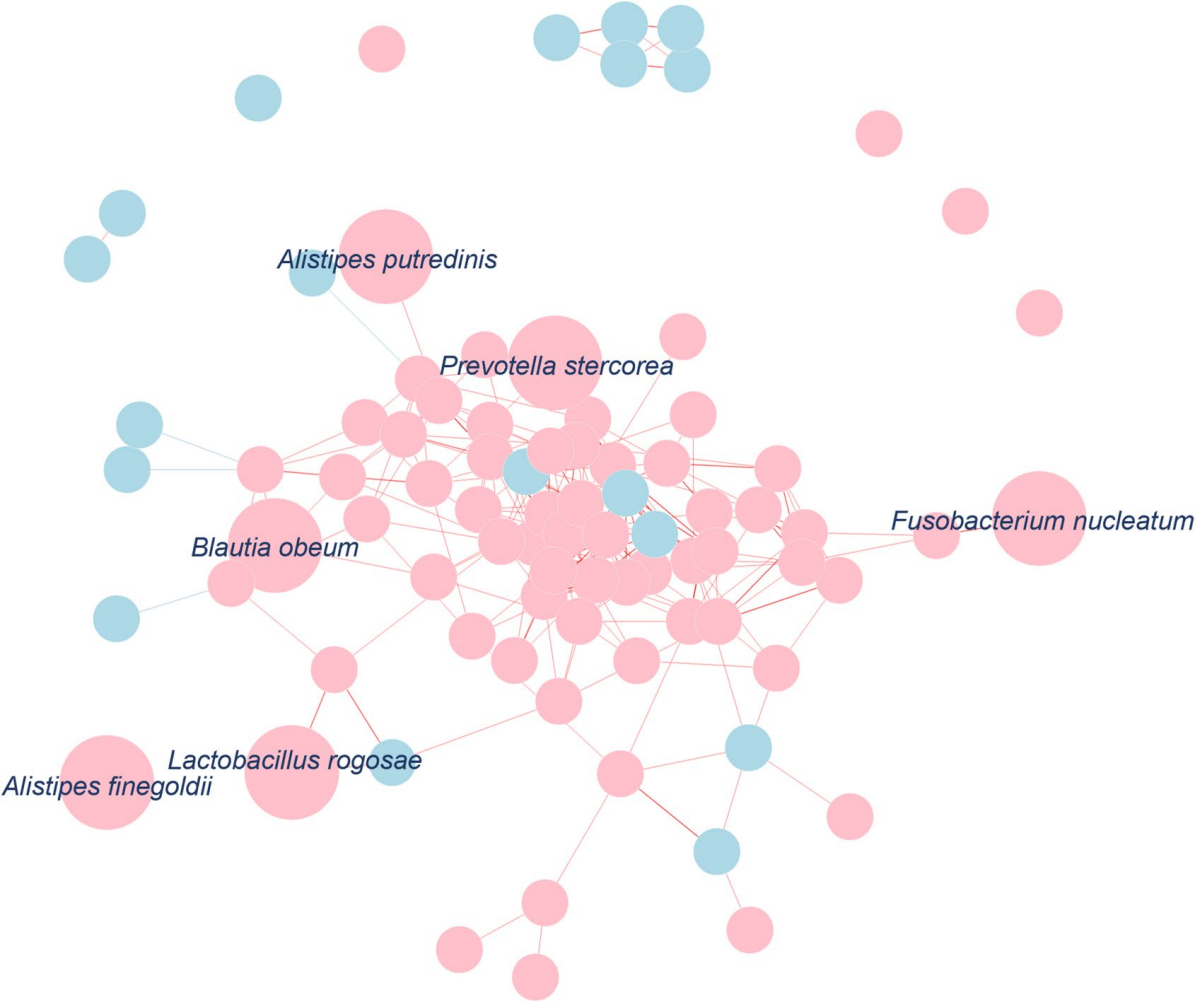
Co-occurrence network of differentially abundant bacterial taxa between HMCC and LMCC. The network was constructed using Spearman’s rank correlation coefficients (p < 0.001) among the 84 bacterial species that showed significant differences in relative abundance between HMCC and LMCC. Nodes represent bacterial species, with pink indicating HMCC-enriched species and blue indicating LMCC-enriched species. Edges denote statistically significant correlations, with red lines for positive and blue lines for negative correlations. Line thickness reflects correlation strength. Six representative species (*Fusobacterium nucleatum, Alistipes putredinis, Prevotella stercorea, Blautia obeum, Lactobacillus rogosae, and Alistipes finegoldii*) are labeled and enlarged for emphasis; node size does not correspond to quantitative values

**Fig. 3:**
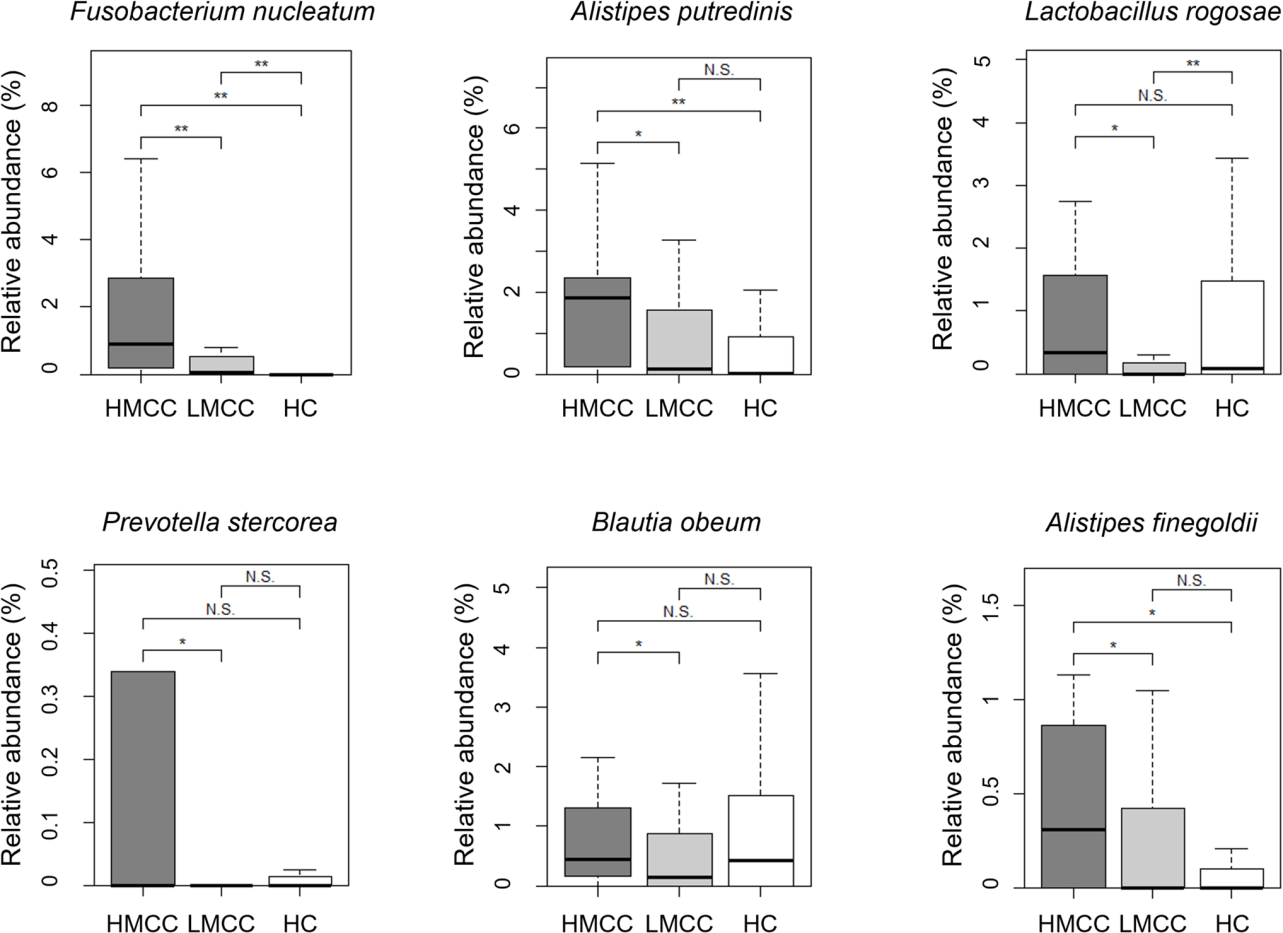
Boxplots illustrating the relative abundance of some significant bacteria; *Alistipes putredinis, Prevotella stercorea, Lactobacillus rogosae, Alistipes finegoldii, Blautia obeum,* and *Fusobacterium nucleatum*; relative abundances are compared among HMCC, LMCC, and healthy control groups; HC, healthy controls; *, p < 0.05; **, p < 0.01

The 16S rRNA gene sequencing of fecal samples from all patients was performed to clarify the gut microbial composition. The dominant phyla in both the HMCC and LMCC groups were *Firmicutes* and *Bacteroidetes*, with *Proteobacteria* and *Actinobacteria* as the third most abundant phyla (Fig. 1A). Shannon index of α diversity (p = 0.07, Fig. 1B) and Bray–Curtis distance of β diversity (p = 0.32, Fig. 1C) showed no significant differences between the two groups. At the species level, 84 bacterial species showed significant differences: 66 species were more abundant in HMCC, whereas 18 were more abundant in LMCC (Fig. 1D and Table 2). To further investigate ecological relationships among these differentially abundant taxa, we performed a co-occurrence network analysis using Spearman’s rank correlation (p < 0.001) (Fig. 2). In the network, HMCC-enriched bacteria such as *Prevotella stercorea* and *Blautia obeum* showed ecological connectivity, forming clusters of positively correlated species. In contrast, *F. nucleatum* appeared relatively isolated, suggesting it may function independently within the HMCC-associated microbiota. Subsequently, the relative abundances of representative bacteria were compared among HMCC, LMCC, and healthy controls (Fig. 3). *F. nucleatum* demonstrated significant differences between HMCC and LMCC, as well as between patients with CRC and healthy controls. Furthermore, the relative abundance of *F. nucleatum* was significantly associated with tumor size, MMR status, lymphatic invasion, and methylation status (Table 3). To identify independent factors associated with HMCC, we conducted a multivariate logistic regression analysis incorporating clinical variables (tumor location, size, lymphatic invasion, differentiation, MMR status, and BRAF status) and the relative abundances of selected bacterial taxa. The analysis revealed that smaller tumor size (p = 0.0026) and *Alistipes putredinis* abundance (p = 0.0110) were independently associated with HMCC. In contrast, *F. nucleatum* and the other bacterial taxa did not reach statistical significance.

**Table 2:** Relative abundances of the top 20 enriched bacteria with significant differences between HMCC and LMCC

Taxon name	Mean abundance (%)		p value
	HMCC (n = 21)	LMCC (n = 65)	
*Alistipes putredinis*	0.99	0.42	0.01
*Prevotella stercorea*	0.94	0.2	0.03
^a^*PAC002209_s*	0.6	0.16	0.02
*Alistipes finegoldii*	0.26	0.16	0.04
^a^*PAC001031_s*	0.48	0.076	< 0.01
^a^*PAC001153_s*	0.71	0	0.01
^a^*PAC001057_s*	0.038	0.2	0.01
*Lactobacillus rogosae*	0.19	0.14	0.02
*Blautia obeum*	0.16	0.14	0.04
^a^*PAC001276_s*	0.34	0.053	< 0.01
^a^*PAC001033_s*	0.34	0.043	0.01
*Porphyromonas_uc*	0.0032	0.11	0.03
^a^*DQ800172_s*	0.13	0.048	< 0.01
^a^*PAC001597_s*	0.061	0.069	< 0.01
^a^*PAC000194_s*	0.11	0.052	0.01
*Fusobacterium nucleatum*	0.089	0.054	< 0.01
*Acutalibacter_uc*	0.00054	0.082	< 0.01
^a^*NFLJ_s*	0.061	0.063	< 0.01
^a^*PAC001129_s*	0.13	0.04	0.03
*Bacteroides rodentium*	0.15	0.029	0.04

^a^Indicates no unique name assigned

**Table 3:** Association between the relative abundance of *F. nucleatum* and clinicopathological factors in 86 CRC patients

	Relative abundance of *F. nucleatum* (%)	
Characteristics	Median (25th–75th percentile)	p value
Methylation status		
HMCC	0.033 (0.0070–0.10)	< 0.01
LMCC	0.0023 (0–0.019)	
Age, year		
≤ 50	0.038 (0.018–0.14)	0.03
> 50	0.0045 (0–0.024)	
Sex		
Male	0.0052 (0–0.025)	0.63
Female	0.0043 (0–0.053)	
Tumor location		
Right	0.012 (0–0.053)	0.19
Left	0.0045 (0–0.023)	
Size, mm		
≤ 50	0.0023 (0–0.018)	< 0.01
> 50	0.027 (0.0064–0.093)	
Histologic differentiation		
Differentiated	0.0052 (0–0.024)	0.84
Undifferentiated	0.0047 (0–0.059)	
Depth of invasion^a^		
Tis + T1 + T2	0.0031 (0–0.017)	0.05
T3 + T4	0.0077 (0–0.054)	
LN metastasis		
No	0.0044 (0–0.018)	0.13
Yes	0.0077 (0–0.057)	
Stage^a^		
0 + I + II	0.0044 (0–0.020)	0.18
III + IV	0.0070 (0–0.055)	
Lymphatic invasion		
No	0.0023 (0–0.019)	0.03
Yes	0.015 (0–0.057)	
Venous invasion		
No	0.0022 (0–0.017)	0.28
Yes	0.0052 (0–0.030)	
MMR status		
pMMR	0.0044 (0–0.024)	0.02
dMMR	0.050 (0.0053–0.15)	
*RAS* status		
Wild type	0.0021 (0–0.018)	0.03
Mutant	0.017 (0–0.049)	
*BRAF* status		
Wild type	0.045 (0–0.024)	0.07
Mutant	0.066 (0.030–0.10)	
Use of probiotics		
Yes	0.043 (0.023–0.43)	0.05
No	0.0047 (0–0.026)	
Use of antibiotics		
Yes	0.12 (0–0.37)	0.57
No	0.0050 (0–0.028)	

*HMCC* high-methylated colorectal cancer, *LMCC* low-methylated colorectal cancer, *MMR* mismatch repair gene function, *pMMR* proficient MMR, *dMMR* deficient MMR

^a^Based on TNM classification

### qPCR for *F. nucleatum*

**Fig. 4:**
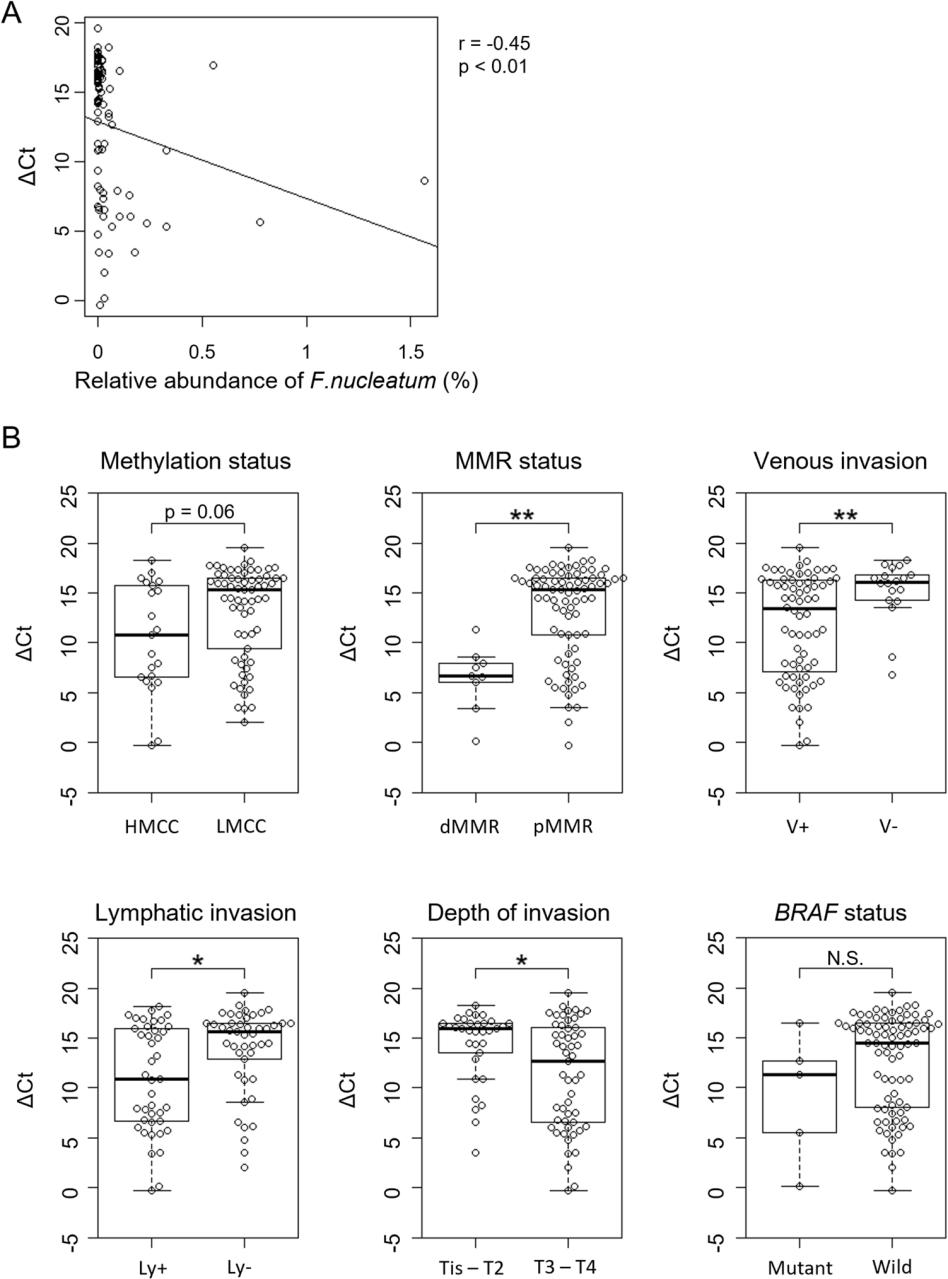
qPCR for *F. nucleatum*. **A** Spearman correlation analysis showing a significant correlation between the relative abundance of *F. nucleatum* in the fecal analyses and the delta Ct value of *nusG*, *F. nucleatum* specific gene (r = −0.45, p < 0.01); **B** comparison of the delta Ct values of *nusG* between the two groups classified by DNA methylation status, mismatch repair gene function (MMR), lymphatic invasion, venous invasion, BRAF status, and pT (tumor invasion); lymphatic and venous invasion and pT were according to the TNM classification; significant differences were found in the grouping using MMR, venous invasion, tumor depth, and lymphatic invasion; Ly+, positive lymphatic invasion; Ly−, negative lymphatic invasion; V+, positive venous invasion; V−, negative venous invasion; *, p < 0.05; **, p < 0.01

To confirm whether the microbial differences associated with DNA methylation status reflected the actual peritumoral environment, qPCR for *F. nucleatum* was performed using genomic DNA from tumor specimens. The ΔCt value of *nusG* normalized with *PGT* had a significant negative correlation with the relative abundance of *F. nucleatum* (ρ = −0.45, p < 0.001, Fig. 4A), indicating a positive correlation between its abundance in tumor tissues and feces. The ΔCt value tended to be lower in HMCC, however, no significance was observed using the Mann–Whitney-U test (p = 0.06, Fig. 4B). The investigation of other factors revealed a significant enrichment of *F. nucleatum* in the dMMR, positive venous and lymphatic invasion, and tumor depth (Fig. 4C–H).

## Discussion

Although recent research has highlighted the role of epigenetic changes in bacteria-induced CRC, studies are limited. We evaluated the characteristics of gut microbiota in HMCC, which is considered a more comprehensive DNA methylation trait than CIMP. As the clinicopathological characteristics of HMCC are unique, a significant cohort was identified. Our gut microbial analyses revealed significant differences in 84 bacterial species, including *F. nucleatum*, between HMCC and LMCC. The load of *F. nucleatum* in CRC specimens significantly correlated with the relative abundance of *F. nucleatum* and tended to be enriched in HMCC tissues. The characteristics of the gut microbiota in HMCC suggest that *F. nucleatum* may be involved in the carcinogenesis of HMCC via DNA methylation. However, further studies are required to determine whether the microbiome acts as a promoter or bystander in this process.

We isolated HMCC using genome-wide DNA methylation status and identified the unique clinicopathological features of HMCC. These results were consistent with our previous reports targeting metastatic CRC using the same classification scheme [24, 27]. As this cohort included non-metastatic CRC, the clinicopathological features of HMCC were independent of stage. Although similar features have been observed in CIMP-positive cases [15, 20, 28], our classification enhanced the precision in identifying characteristic populations. The clinicopathological features of HMCC include poor prognostic factors, such as *BRAF* mutation, dMMR, and undifferentiated histology. Given the distinctly different characteristics of HMCC from those of LMCC, the development of therapies and prevention strategies targeting HMCC is desired.

Our analysis of the gut microbial composition via 16S rRNA gene sequencing revealed *Firmicutes* and *Bacteroidetes* as the two predominant phyla in both HMCC and LMCC groups. These two phyla are enriched in both fecal and tissue samples from patients with CRC and healthy adults [29–31], suggesting that the analysis was appropriately performed. No significant differences in microbial composition were found between HMCC and LMCC, at least in general patterns. However, 84 bacteria with varying relative abundances were detected upon individual comparison. The identified bacteria included *F. nucleatum*, one of the most well-known bacteria associated with CRC [32]. Increased levels of *F. nucleatum* are reported in advanced CRC [33, 34]. Furthermore, its association with CIMP, MSI-H, and *BRAF* mutation has also been revealed [35–37].

The results of the present study, in which *F. nucleatum* was more abundant in HMCC than in LMCC and in patients with CRC than in healthy controls, are consistent with previous studies. Xia et al. showed that *F. nucleatum* increases the expression of DNA methyltransferases and induces promoter hypermethylation of several tumor suppressor genes, resulting in tumorigenesis [38]. Additionally, an increased abundance of *F. nucleatum* is associated with a poor prognosis of CRC. Large numbers of *F. nucleatum* also correlate with factors similar to HMCC, such as MSI, CIMP, dMMR, and proximal location [36, 39–41]. The HMCC cases in this study were related to undifferentiated histology, lymphatic invasion—suggesting poor prognosis—and *F. nucleatum* concentration. Therefore, it is possible that *F. nucleatum* may not be a direct prognostic factor, but rather that HMCC induction by *F. nucleatum* is the essence of its prognostic involvement.

Nevertheless, it remains unclear whether *F. nucleatum* directly contributes to HMCC development or preferentially proliferates within the altered tumor microenvironment. Although previous studies suggest *F. nucleatum* may influence DNA methylation, changes in mucosal immune responses and epithelial surface properties associated with tumor hypermethylation could also facilitate its colonization. Thus, *F. nucleatum* may act as either a promoter or a bystander in HMCC development. Future studies using experimental models, such as co-culture systems of colorectal epithelial cells with *F. nucleatum* and gnotobiotic mouse models, are needed to clarify this causal relationship. Furthermore, our multivariate analysis, which included clinical factors and representative bacterial taxa, showed that smaller tumor size and *Alistipes putredinis* abundance were independently associated with HMCC status, whereas *F. nucleatum* did not reach statistical significance. These findings suggest that HMCC development may be influenced by a combination of clinical and microbial factors rather than by a single dominant bacterium. Additionally, given the complex microbial alterations in HMCC, the potential contribution of other bacterial species beyond *F. nucleatum* warrants further investigation.

If *F. nucleatum* is causally linked to HMCC development, several therapeutic strategies could target this bacterium. Antibiotics, particularly metronidazole, have been shown to reduce *F. nucleatum* burden in colorectal tumors and suppress tumor growth in mouse models [42]. Additionally, modulating the gut microbiota through probiotics, prebiotics, or dietary interventions may help control *F. nucleatum* colonization indirectly. However, clinical evidence demonstrating that reducing *F. nucleatum* burden alters DNA methylation patterns or improves patient outcomes is lacking. Further studies are warranted to assess whether targeted modulation of *F. nucleatum* can impact the epigenetic landscape and clinical progression of HMCC.

Furthermore, the analysis of *F. nucleatum* levels in tumor tissues revealed a significant correlation between the ΔCt value of *nusG* and the relative abundance of *F. nucleatum* in fecal samples. The results showing that *F. nucleatum* tended to be enriched in HMCC tissues are consistent with those of previous reports [28, 37]. *F. nucleatum* and *Peptostreptococcus anaerobius* play key roles in CRC development by adhering to mucosal cells through specialized membrane proteins [43, 44]. This suggests that *F. nucleatum* promotes DNA methylation via a cell adhesion-based mechanism. Although *F. nucleatum* was significantly enriched in HMCC and has been implicated in promoting DNA methylation and tumorigenesis, HMCC development likely involves additional contributing factors. Our multivariate analysis revealed that smaller tumor size and *Alistipes putredinis* abundance were independently associated with HMCC, whereas *F. nucleatum* did not reach statistical significance after adjusting for clinical factors. This suggests that HMCC development may be influenced by a combination of clinical and microbial factors, rather than being driven by a single dominant bacterium.

Thus, beyond *F. nucleatum*, other bacterial species and alterations in the gut microbial community structure may play important roles in HMCC pathogenesis. *Prevotella stercorea* and *Alistipes finegoldii* have been linked to CRC development [45, 46], and *Blautia obeum* is more enriched in CRC tissues than in normal mucosal tissues [47]. However, the specific mechanisms through which they affect CRC remain unclear. To explore ecological relationships among differentially abundant bacterial taxa, we constructed a co-occurrence network based on correlation patterns. This analysis revealed that certain HMCC-enriched species, such as *Prevotella stercorea* and *Blautia obeum*, formed clusters of positively correlated bacteria, suggesting possible cooperative behavior. In contrast, *F. nucleatum* appeared relatively isolated, supporting the notion that it may function independently in HMCC pathogenesis. These findings underscore the complexity of microbial interactions and the need for integrative analyses in future studies.

This study had some limitations. First, the gut bacterial analysis relied primarily on fecal samples without comprehensive tissue analysis. However, significant differences in bacterial abundance between the two groups were identified, with *F. nucleatum*, a bacterium linked to CRC and DNA methylation, being the most prevalent in HMCC. This result is consistent with those of previous reports. Second, due to the limited sample size, we were unable to perform propensity score matching to adjust for baseline characteristics between HMCC and LMCC. Given the significant differences in patient background between these groups, larger cohort studies are needed to validate our findings and clarify the role of the gut microbiota. Third, this study lacked functional validation through experiments such as co-culturing colon cancer cells with bacteria or administering bacterial metabolites. Therefore, the specific mechanisms underlying these bacterial effects remain unclear. Furthermore, we did not experimentally investigate whether suppressing *F. nucleatum* modulates DNA methylation patterns. Future studies are warranted to clarify this causal relationship. Additionally, it cannot be ruled out that differences in the gut microbiota composition result from the presence of HMCC rather than contribute to it. However, further investigation is required to resolve these issues.

In conclusion, we revealed the characteristics of the gut microbiota in HMCC. Although further studies are needed, modulating gut microbiota composition may help prevent HMCC and improve prognosis, given the reversible nature of DNA methylation.

## Data Availability

The clinical datasets used in the current study are not publicly available due to participant privacy considerations; however, they can be obtained from the corresponding author upon reasonable request. The 16S rRNA sequencing data have been deposited in DDBJ under the BioProject accession number PRJDB20352.
